# Patients' preferences for adjuvant endocrine therapy in early breast cancer: what makes it worthwhile?

**DOI:** 10.1038/sj.bjc.6602874

**Published:** 2005-12-06

**Authors:** V M Duric, L J Fallowfield, C Saunders, J Houghton, A S Coates, M R Stockler

**Affiliations:** 1 NHMRC Clinical Trials Centre, University of Sydney, Sydney, Australia; 2 Cancer Research UK, Psychosocial Oncology Group, University of Sussex, Falmer, Brighton, East Sussex, BN1 9QG, UK; 3 University Department of Surgery, Royal Perth Hospital, Perth, Australia; 4 Clinical Trials Group, Department of Surgery, Royal Free and University College Medical School, London, UK; 5 School of Public Health, University of Sydney and The Cancer Council, Sydney, Australia; 6 Sydney Cancer Centre – RPA and Concord Hospitals, Sydney, Australia

**Keywords:** adjuvant endocrine therapy, early breast cancer, patient preferences

## Abstract

Adjuvant endocrine therapy improves recurrence and survival rates, but has side effects and is inconvenient. The aim of this study was to determine the preferences of premenopausal women who had adjuvant endocrine therapy in a randomised trial. In all, 85 (or eighty-five) women completed semistructured interviews 6–30 months after finishing adjuvant endocrine therapy. Hypothetical scenarios based on known potential survival times (5 or 15 years) and rates (60% or 80% at 5 years) without adjuvant endocrine therapy were used to determine the smallest gains women judged necessary to make their adjuvant endocrine therapy worthwhile. Although a third of the women considered gains of 1% in survival rates or 6 months in survival times sufficient to make their adjuvant endocrine therapy worthwhile, more than half the women required gains of at least 5% in survival rates or 3 years in survival time as necessary to make adjuvant endocrine therapy worthwhile. Larger benefits were required by women who had longer treatment, worse side effects, and by those who were treated with goserelin alone. The route of administration (tablet *vs* injection) did not affect preferences and some women judged small benefits sufficient to make their adjuvant endocrine therapy worthwhile, but many women required larger benefits than their counterparts in similar studies of preferences for adjuvant chemotherapy.

Adjuvant endocrine therapy reduces recurrence rates and improves overall survival in women with hormone-receptor positive early breast cancer. The relative effects of these treatments seem to be independent of nodal status, menopausal status, age, and use of adjuvant chemotherapy. The absolute benefits for an individual woman depend on her baseline risk of recurrence. The Oxford Overviews suggest that 5 years of adjuvant tamoxifen improves 10-year survival rates by 5–11%, and that ovarian ablation improves 10-year survival rates by 6–13%. (Early Breast Cancer Trialists’ Collaborative Group, 1998, 2005).

Adjuvant endocrine therapy has side effects that may include a wide range of menopausal symptoms, infertility, and osteoporosis. The side effects of endocrine therapy may be less intense than those of chemotherapy, but they are perhaps more important to women than clinicians realise (Fellowes *et al*, 2001). While adjuvant endocrine therapy is of undoubted benefit to a group of women with hormone-receptor positive breast cancer, it is unnecessary for some individuals in the group because their cancer would not have recurred in any case, and it is ineffective for others whose cancer would recur just as early with adjuvant endocrine therapy as without it. The effects of various combinations of ovarian ablation, chemotherapy, tamoxifen, and aromatase inhibitors in younger women with hormone-receptor positive early breast cancer are the subject of ongoing international randomised trials (Dellapasqua *et al*, 2005).

We aimed to determine the benefits judged necessary to make adjuvant endocrine therapy worthwhile by women who participated in a large randomised clinical trial evaluating 2 years of adjuvant endocrine therapy with tamoxifen, goserelin, or both in women 50 years or younger with early breast cancers that expressed hormone receptors (the Under Fifties Trial) (Dellapasqua *et al*, 2005). This trial was of a pragmatic design, allowing a range of local treatments and adjuvant chemotherapy if required. Measuring the preferences of patients who have had a treatment gives insights into how they trade-off the treatment's benefits and harms. Many women with early breast cancer judge small benefits sufficient to make adjuvant chemotherapy worthwhile despite its inconvenience and side effects (Duric and Stockler, 2001; Jansen *et al*, 2001; Duric *et al*, 2005). The benefits of adjuvant endocrine therapy in women with hormone-receptor positive breast cancer are comparable to those of adjuvant chemotherapy. Most women who had adjuvant endocrine therapy as part of routine clinical practice judged modest gains necessary to make adjuvant endocrine therapy worthwhile (Thewes *et al*, 2005). How comparable are these benefits to those of premenopausal women who had adjuvant endocrine therapy for early breast cancer as part of a randomised trial?

## PATIENTS AND METHODS

### Participants

Women in the Under Fifties trial had invasive, operable early breast cancer that had already been treated with standard surgery, with or without adjuvant radiotherapy, and with or without adjuvant chemotherapy. Eligible consenting women were randomised to one of four arms:

1. Observation without adjuvant endocrine therapy;
2. Tamoxifen 20 mg daily by mouth for 2 years;
3. Goserelin 3.6 mg monthly by subcutaneous injection for 2 years and;
4. Both tamoxifen 20 mg daily and goserelin 3.6 mg monthly for 2 years.

New information about the benefits of tamoxifen for women under 50 years became available from the Oxford Overviews during recruitment and investigators were subsequently given the option of limiting randomisation to two of the arms by prescribing tamoxifen and then randomising whether goserelin was given. Most women in the current preferences study were recruited in the latter years of the trial when tamoxifen was prescribed to most women with hormone-receptor positive tumours (rather than randomly allocated).

We approached 120 women in the Under Fifties Trial from 10 UK hospitals who completed their adjuvant endocrine therapy 6–30 months previously (and 30–60 months from trial entry): 88 agreed and 85 (71%) were interviewed. The median time from trial entry to interview was 36 months. The reasons for nonparticipation were the following: declined (17), no response or lost to follow-up (16), agreed but did not attend (3). Women gave separate, written informed consent for the preferences study.

### Preferences

During a semistructured interview, participants answered a series of questions about hypothetical clinical scenarios based on those developed by Simes and Coates (2001) to survey preferences for adjuvant chemotherapy in early breast cancer. Two different types of questions were asked: a ‘survival time trade-off’ based on increasing a given length of survival time; and a ‘survival rate trade-off’ based on increasing the probability of surviving a given length of time. Participants identified the smallest improvement in survival time or survival rate they judged necessary to make adjuvant endocrine therapy worthwhile. Women who had endocrine therapy were asked to base their judgements on their own experience. Women who did not have adjuvant endocrine therapy (seven out of 85, 8%) were given a list of the common side effects of adjuvant endocrine therapy. Liaison during study design ensured that methods were closely similar to those used by Simes and Coates (2001).

For the survival time trade-off, participants considered a baseline survival of 5 years without adjuvant endocrine therapy, and were then asked whether they would prefer 5 years survival without adjuvant endocrine therapy, or a longer survival time with adjuvant endocrine therapy, with increments ranging from 1 extra day to 20 extra years. The same process was used for the scenario with an expected survival time of 15 years except that the maximum benefit was 30 extra years. For the survival probability trade-off, participants considered baselines of either a 60% or an 80% 5 year survival rate without adjuvant endocrine therapy, and were then asked to indicate whether they would prefer the baseline survival rate without adjuvant endocrine therapy, or a higher survival rate with adjuvant endocrine therapy, with increments ranging from 1% to the maximum possible benefit of 20% for the 80% baseline and 40% for the 60% baseline. A sliding ruler was used to illustrate the trade-offs. The slide was moved back and forth to show different benefits until the participant identified the minimum benefit she considered necessary to make adjuvant endocrine therapy worthwhile.

The effects of changing various aspects of treatment were tested by giving women hypothetical scenarios based on the 15 year survival time scenario, and then increasing the duration of therapy from 2 years to 5 years, or omitting the worst side effect, or changing the route of administration for goserelin from injection to tablet, or for tamoxifen from tablet to injection. We also asked women to consider the minimum benefit needed to make adjuvant endocrine therapy worthwhile if it was for her sister or best friend.

To address framing effects, the presentation of benefits was randomised to either start with the smallest benefit and then increase in magnitude, or start with the largest benefit and then decrease in magnitude.

Interviews lasted about 30 min. All interviewers were trained at a 2-day course, and/or individually. Explanatory qualitative data were transcribed from tape (two interviews were not tape recorded). Taped interviews were checked to provide feedback to interviewers. Interviewers recorded their perceptions of the participant's level of understanding and distress during the interview.

Data were also collected on the frequency and severity of side effects attributed to systemic therapy for breast cancer (chemotherapy and/or endocrine therapy). Women were asked to attribute their side effects to a specific treatment. Women also answered questions about their marital status, employment, children, and dependants.

### Statistical methods

The aims of this study were largely descriptive. The statistical methods were based on those used in similar previous studies (Simes and Coates, 2001; Duric *et al*, 2005). Preference data were described with graphs of the proportions of women judging various benefits sufficient to make adjuvant endocrine therapy worthwhile. The effects of baseline and study factors used the sum of the two survival time scenarios as the primary outcome variable to avoid the problems of multiple outcome measures.

Rater effects were tested by comparing the preferences elicited by different interviewers using the nonparametric Kruskal–Wallis test for multiple independent samples. Framing effects were tested by comparing preferences elicited by starting with the smallest benefit with those elicited by starting with the largest benefit using the nonparametric Mann–Whitney test for two independent samples. Differences in preferences according to hypothetical variations in treatment duration, side effects, and delivery were tested with the nonparametric Wilcoxon signed rank test for paired samples. Differences in preferences according to whether therapy was intended for the patient or her sister/best friend were also tested with the Wilcoxon signed rank test for paired samples.

Associations between preferences and baseline characteristics were assessed with linear regression models after applying the normal score transformation to the sum of the two survival time scenarios. Associations with the following characteristics were assessed: types of adjuvant systemic therapy (i.e. tamoxifen, goserelin and chemotherapy), recurrence of cancer, having children, having a life partner, being employed, age, and time from randomisation in the clinical trial. The association of each baseline characteristic with preferences was first tested on its own (univariable analysis) and then together with other baseline characteristics (multivariable analysis) (Katz MH, 1999).

SPSS for Windows, version 11.5 was used for all analyses.

## RESULTS

The median age of women was 45 years at the time of interview (range 31–54). Most were married or living with a partner (84%), and most had children (88%). Most had tamoxifen, either in combination with goserelin (50%) or alone (35%); few had goserelin alone (8%) or no adjuvant endocrine therapy (7%). Breast cancer had recurred in four out of 85 women. About a third of the women also had adjuvant chemotherapy (35%).

The most commonly reported endocrine symptoms were hot flushes (91%), weight gain (80%), sweats (52%) and fatigue (50%). Women attributed most of their endocrine symptoms to adjuvant endocrine therapy (range 81–93%). Frequency and severity of side effects were rated highest by patients who had the combination of tamoxifen and goserelin (median 19, range 0–83); less with goserelin alone (median 18, range 2–29); and least for tamoxifen alone (median 12, range 2–34).

There were no significant effects of interviewers (*P*=0.2) or framing (*P*=0.9) on preferences. Interviewers judged that most women (87%) understood the purpose of the interview and that most women (84%) had little or no difficulty understanding the preference questions. Interviewers judged that few women (5%) were distressed by the interview.

**Figure 1:**
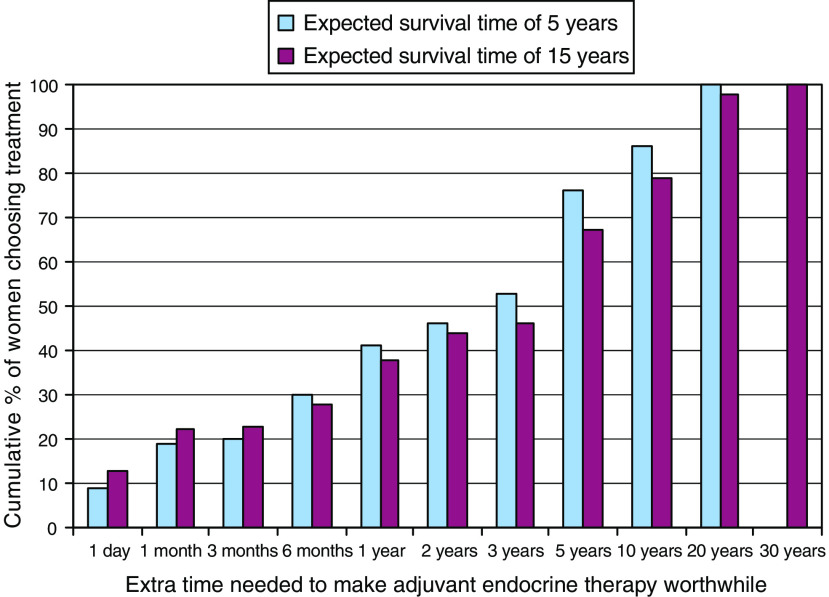
Cumulative proportions of women considering whether adjuvant endocrine therapy would be worthwhile for varying increments in 5 and 15 year expected survival time (*N*=85).

**Figure 2:**
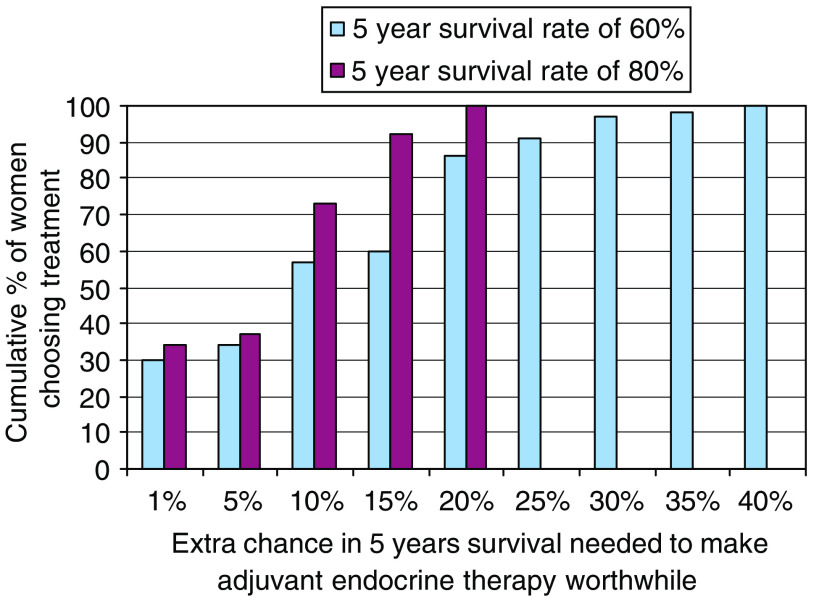
Cumulative proportions of women considering whether adjuvant endocrine therapy would be worthwhile for varying increments in 5 year survival rate of 60 and 80% (*N*=85).

Women's preferences were highly variable, and while some judged small benefits sufficient to make adjuvant endocrine therapy worthwhile, many required much larger benefits as necessary (see Figures 1 and 2). About a third of women judged gains of 1% in survival rate or 6 months in survival time sufficient to make adjuvant endocrine therapy worthwhile. More than half judged gains of at least 5% or 3 years necessary, and about one in six judged gains of at least 15% or 10 years necessary to make their adjuvant endocrine therapy worthwhile.

Changes to various aspects of the hypothetical scenarios affected women's preferences. A median gain of 5 years was judged necessary to make 2 years of adjuvant endocrine therapy worthwhile on a baseline of 15 years without adjuvant endocrine therapy. A larger gain was judged necessary if treatment lasted 5 years instead of 2 years (median 7 years, *P*<0.0001). A smaller gain was judged sufficient if the worst side effect of adjuvant endocrine therapy was removed (median 4 years, *P*=0.005). Preferences were not affected by hypothetical changes in the route of administration (for goserelin: from injection to tablets, *P*=0.3; and, for tamoxifen: from tablet to injection, *P*=0.4). There was a weak trend towards larger gains being judged necessary if the endocrine therapy was being recommended to a sister or best friend (median gain of 10 *vs* 5% on a baseline of 80% 5 year survival without adjuvant endocrine therapy, *P*=0.07).

The effects of baseline characteristics on preferences are summarised in Table 1. Only two factors were significantly associated with preferences, and both were significant either alone in separate models, or together in the same model. Smaller benefits were judged sufficient by women who had fewer side effects, and by women who had tamoxifen (either alone or with goserelin). Preferences for adjuvant endocrine therapy were not associated with time from trial entry to interview, having adjuvant chemotherapy, recurrence of breast cancer, or demographic factors, although there was a weak trend towards employed women judging smaller benefits sufficient (*P*=0.08).

## DISCUSSION

A third of the women judged small gains sufficient to make adjuvant endocrine therapy worthwhile, but over half the women judged much larger benefits necessary. This is surprising given the widespread perception by healthcare professionals that endocrine therapy has only modest toxicity.

Women who have had adjuvant chemotherapy for early breast cancer judge small benefits sufficient to make it worthwhile, despite its significant side effects and inconvenience (Duric and Stockler, 2001; Jansen *et al*, 2001; Duric *et al*, 2005). Adjuvant endocrine therapy provides comparable benefits for women with hormone-receptor positive disease, and is generally considered to do so with less severe side effects and less inconvenience. More than half the women who had adjuvant endocrine therapy as part of routine clinical practice judged 2% gain in survival rate or an additional 3–6 months sufficient to make adjuvant endocrine therapy worthwhile (Thewes *et al*, 2005). Yet women in this study required larger benefits to make adjuvant endocrine worthwhile than those judged necessary to make chemotherapy worthwhile in comparable studies using almost identical methods (Duric and Stockler, 2001; Jansen *et al*, 2001; Duric *et al*, 2005) and larger still than those required by women who had endocrine therapy as part of routine clinical practice (Thewes *et al*, 2005).

Our study identified several factors that were associated with the size of the benefit required to make adjuvant endocrine therapy worthwhile. Longer duration of therapy and greater toxicity attributed to therapy were associated with a requirement for larger improvements. Women who had tamoxifen judged smaller benefits necessary to make adjuvant endocrine therapy worthwhile, suggesting that tamoxifen was better tolerated than goserelin. However, the route of administration seemed to matter less than perceived side effects.

When asked to make similar judgements for their close relative or best friends, most women judged that greater benefits would be necessary to make adjuvant endocrine therapy worthwhile for the other woman.

Despite the similar methods of the two studies, approximately half the women in the Thewes *et al* (2005) judged an extra 3 months sufficient compared to women in the current study who judged an extra 3 years necessary to make adjuvant endocrine therapy worthwhile. A similar difference occurred on the survival rate scenarios, with about three-quarters of the women in the Thewes *et al* (2005) study judging a 5% improvement sufficient to justify endocrine therapy, compared to about a third of the women in our current study. However, about two-thirds of the women in the Thewes *et al* (2005) study were treated with tamoxifen alone compared to about a third of the women in the current study. In the current study, women who had goserelin alone judged larger benefits necessary than women who had tamoxifen alone or goserelin with tamoxifen. This suggests that tamoxifen may reduce the side effects of goserelin.

All the women in the Thewes *et al* (2005) study had chemotherapy as part of routine clinical practice compared to the current study which was performed within the context of a clinical trial. Whether trial participation and type of treatment are factors involved in the apparent differences in preferences between the two studies needs to be explored in future research.

Studies of preferences for adjuvant chemotherapy in early breast cancer found that less severe side effects and having dependents were associated with smaller benefits being judged sufficient to make it worthwhile (Duric and Stockler, 2001; Duric *et al*, 2005). Severity of side effects was similarly associated with preferences in this study, but having dependents was not significantly associated with preferences in this study, perhaps because of the greater homogeneity of our sample and the small number of women without dependents. We were surprised not to find an effect of age. Future studies should explore the importance of fertility and related issues to younger women considering adjuvant endocrine therapy.

Our study has limitations. Our sample included women participating in a randomised trial who may not be representative of women in general. Our sample included a mixture of women who had goserelin, tamoxifen, or both. The few women who did not have endocrine therapy based their judgements on information about side effects rather than direct experience. Tamoxifen was given electively to some women and was randomly allocated to others, so comparisons between women who did and did not have tamoxifen may be affected by selection bias. About a third of the women had chemotherapy also and distinguishing between the side effects of the various treatments would have been difficult. This kind of research has other limitations that have been discussed in detail previously (Duric *et al*, 2005). Preferences were based on women's recollections of what treatment was like, and were likely to be influenced by psychological mechanisms, including memory, adaptation, coping, and cognitive dissonance reduction. Furthermore, because only women who had adjuvant endocrine therapy were recruited, they are not representative of all women considering whether to have adjuvant endocrine therapy.

Endocrine therapy and chemotherapy are very different treatments. The side effects of endocrine therapy are generally considered milder but are longer lasting than those of chemotherapy. The finding that women having adjuvant endocrine therapy judged larger benefits necessary to make it worthwhile than women having adjuvant chemotherapy is striking. It suggests that shorter treatments are easier to cope with even if they have more severe side effects. However, comparisons of our results with those from studies of women having adjuvant chemotherapy should be interpreted cautiously because, although the methods were very similar, they were not identical and the populations were different. Women in our study were younger, and about a third had already had chemotherapy.

Studies of preferences offer valuable information to clinicians, patients, and communities about the benefits patients require to make treatments worthwhile. The benefits required to make adjuvant endocrine therapy worthwhile were larger than we expected suggesting that the side effects of adjuvant endocrine therapy were more important to women than we expected. Clinicians should ask women about their circumstances, priorities, and concerns because these may influence their choices about treatment. Research is also needed to determine the best way to elicit and incorporate this kind of information in clinical decision-making.

## Figures and Tables

**Table 1:** Factors associated with judging smaller benefits sufficient to make adjuvant endocrine therapy worthwhile either alone (univariable analysis) or together (multivariable analysis)

	**Univariable analysis *P***	**Multivariable analysis *P***
*Demographic factors*		
Age	0.15	
Whether she		
Was employed at the time of interview	0.08	
Has a life partner	0.75	
Has any dependant children	0.65	
		
*Treatment factors*		
Less side effects from hormonal treatments	0.02	0.02
Stopped trial treatment early	0.17	
Had tamoxifen	0.03	0.04
Had goserelin	0.57	
Had adjuvant chemotherapy	0.14	
Had a recurrence	0.94	
Time elapsed since treatment	0.29	
